# Occurrence of juvenile *Dermacentor reticulatus* ticks in three regions in Poland: the final evidence of the conquest

**DOI:** 10.1186/s13071-021-05039-z

**Published:** 2021-10-14

**Authors:** Dorota Dwużnik-Szarek, Ewa Julia Mierzejewska, Anna Bajer

**Affiliations:** Department of Eco-Epidemiology of Parasitic Diseases, Institute of Developmental Biology and Biomedical Sciences, Faculty of Biology, University of Warsaw, Miecznikowa 1, 02-096 Warsaw, Poland

**Keywords:** *Dermacentor reticulatus*, *Haemaphysalis concinna*, *Ixodes ricinus*, Larvae, Nymphs, Poland, Rodents

## Abstract

**Background:**

Two populations of *Dermacentor reticulatus* ticks (Western and Eastern) in Poland are among the most dynamic tick populations in Central Europe. Expansion and settlement of ticks in new localizations depend on the presence of suitable hosts, for both adult and juvenile ticks.

**Methods:**

The current study was planned to complement our previous studies on questing adult ticks and was focused on a collection of juvenile *D. reticulatus* ticks from rodents from three regions in Poland, defined by the presence/absence of adult ticks (regions of the Western and Eastern tick population and the gap area between them) to confirm the existence of stable populations. Rodent trapping was conducted in open habitats (fallow lands, wasteland and submerged meadows) in 2016–2018 in June, July and/or August to encompass seasonal peaks of larvae and nymph activity.

**Results:**

Altogether, three tick species were collected, 2866 *D. reticulatus,* 2141 *Ixodes ricinus* and 427 *Haemaphysalis concinna*. *Dermacentor reticulatus* was the most common (72.3%) and abundant (mean 17.94 ± 2.62 ticks/rodent) tick species on rodents from the Eastern region; in the Western region infestation of rodents was only 6.8%. *Ixodes ricinus* was found in all three regions and was the only tick species collected from rodents from the gap area. *Haemaphysalis concinna* was noted only in the Western region. The highest infestation of juvenile *D. reticulatus* was recorded on voles (*Myodes* and *Microtus* spp.), infestation of *I. ricinus* was the highest on *Apodemus* mice, and the majority of *H. concinna* ticks were collected from root voles *Alexandromys oeconomus*.

**Conclusions:**

Our study confirmed a stable population of *D. reticulatus* in Eastern and Central Poland and a lower prevalence and mean abundance of this tick species among rodents from the Western region. A lack of juvenile *D. reticulatus* on rodents in Niewiadów confirmed the existence of the gap area, free of *D. reticulatus* ticks.

**Graphical abstract:**

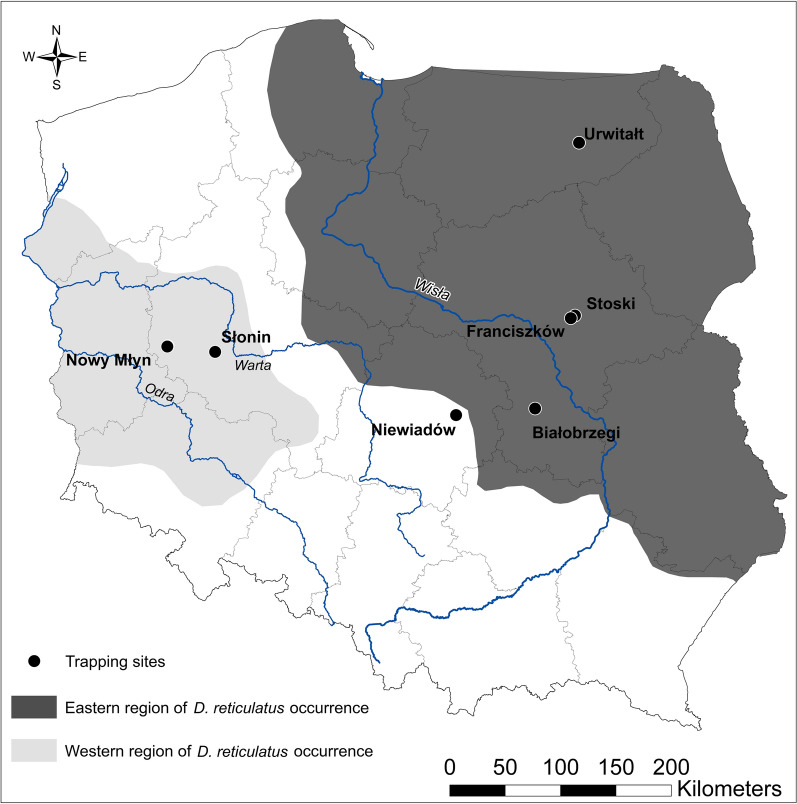

**Supplementary Information:**

The online version contains supplementary material available at 10.1186/s13071-021-05039-z.

## Background

Occurrence of vector-borne diseases depends on the availability of appropriate vectors. In recent years a growing number of papers have reported on the spread of numerous vectors, including a cluster of mosquitoes and tick species in Southern and Central Europe [1–3].

Two populations of *Dermacentor reticulatus* ticks (Western and Eastern) in Poland are among the most dynamic tick populations in Central Europe [4–6]. We have recently documented seasonal and annual changes in the geographical range of both these populations, resulting in a measurable decrease in the size of the area historically free of this tick species (gap) and a continuous spread of canine babesiosis in the area of Poland, particularly in the eastern expansion zone [6]. This study was based on the collection of adult questing *D. reticulatus* ticks from vegetation.

The present study was planned to complement our previous studies on adult ticks, exploring the occurrence of juvenile *D. reticulatus* ticks on rodents in two endemic regions with their respective expansion zones and the gap area. Rodents are an important host for juvenile ticks, including *Ixodes ricinus*, *Haemaphysalis concinna* and *D. reticulatus* [7–9]. Thus, availability of these hosts for larvae and nymphs is necessary for the survival of newly settled ticks in new locations [1, 10, 11]. Larvae and nymphs of *D. reticulatus* are nidiculous and feed mainly on voles (*Microtus* spp. and *Alexandromys oeconomus*) [7, 8, 12, 13]. Without an appropriate host species for the juvenile stages of *D. reticulatus*, completion of a tick life cycle and subsequent progressive colonization of new areas are impossible. Therefore, recording of juvenile *D. reticulatus* ticks on rodents is likely the most convincing evidence for the successful establishment of a stable population at a particular site.

The main aim of this present study was to determine the occurrence of *D. reticulatus* instars on rodents from open habitats in two regions (Eastern and Western) and in the gap area, presumably free of *D. reticulatus*. We expected to witness similar trends as for adult ticks [6]: high rodent infestation with *D. reticulatus* juvenile stages in Eastern Poland (hyperendemic for *D. reticulatus* occurrence with high tick abundance) and lower on rodents in the western part of the country. Since in Central Poland adult *D. reticulatus* ticks still have not been detected, we also expected that rodents from the gap region should be free from *D. reticulatus* instar infestation.

## Methods

The study was performed each summer in the period 2016–2018. Rodent trapping was conducted at each site in June to encompass the seasonal peak of larval *D. reticulatus* activity and then in July and/or August to encompass the seasonal peak of nymph activity [14].

**Fig. 1:**
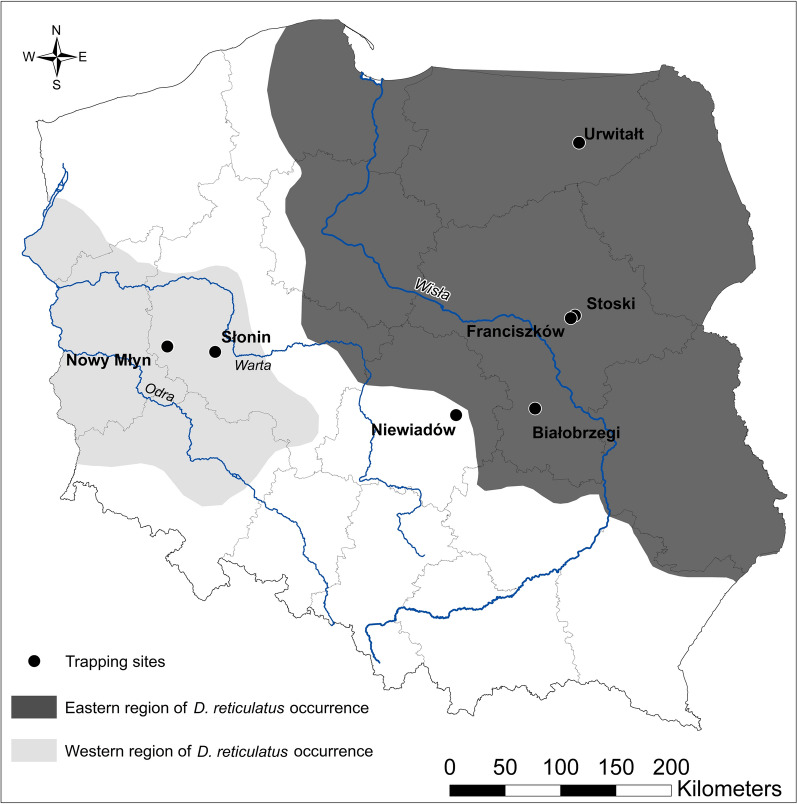
Rodent trapping sites

Altogether, three sites in the area endemic for the eastern population of *D. reticulatus* were monitored in the Mazovia voivodeship—Białobrzegi (51.66445N, 20.94700E); Stoski (52.409226N, 21.509125E); Franciszków (52.388847N, 21.452349E)—and one site in the Warmia-Mazuria voivodeship: Urwitałt (53.809318N, 21.647636E) (Fig. 1).

Two sites were selected in the western region of *D. reticulatus* occurrence: Słonin (52.119201N, 16.747274E) and Nowy Młyn (52.148505N, 16.114098E) in the Greater Poland voivodeship and finally one site in the gap (non-endemic region for *D. reticulatus*), Niewiadów (51.6237N, 19.9150E), in the Łódź voivodeship (Fig. 1). In 2016, two sites from the eastern endemic region were examined (Franciszków and Stoski); in 2017 two sites from the eastern endemic region and one in the gap region (Białobrzegi, Urwitałt, Niewiadów), and finally in 2018, three sites in the eastern region, one in the gap region and two in the western region were examined (Białobrzegi, Franciszków, Stoski, Nowy Młyn, Słonin, Niewiadów).

Trapping sites were located in habitats preferable for *D. reticulatus*, such as meadows, fallow lands and wetlands. The main target constituted voles from the genera *Microtus* and *Alexandromys*, the preferable hosts for larvae and nymphs of *D. reticulatus* [7, 8, 13–15]. All methods for rodent trapping and tick collection were described in detail in our previous papers [7, 8, 16]. In short, traps were set along 2–3 transects about 100 to 300 m long, depending on the topography of the examined site. Transects were from 10 to 20 m distant. One live trap was set at one point in 10-m intervals. Traps were set in the evening and inspected next day in the morning (6–9 a.m.). We used 50 traps during 3 nights at each trapping site. In each site two trapping sessions were conducted, in June or the first days of July and then in the last week of July or the first days of August. The bait used was a mixture of grains (for mice *Apodemus* spp.) and chopped fresh fruits and vegetables (apples, cucumbers, carrots) (for voles *Microtus* and *Alexandromys*). Rodents were live-trapped and live-processed, under light isoflurane anesthesia, and then released near their trapping point. Selected measurements of the rodent body were performed: head length, head width, nose-anus length, tail length and foot length, and weight. Species, age, sex and sex activity were determined as previously described [16, 17]. During the handling, rodents were carefully examined for the presence of ticks. All collected ticks were preserved in 70% ethanol.

Identification of collected ticks to species and stages was made according to a morphological key [18] using a stereoscopic microscope Zeiss Stemi 508 in the Department of Parasitology, Faculty of Biology, UW.

### Statistical analysis

All statistical analyses were conducted in the IBM SPSS Statistics v. 21 software package (IBM Corp.). For the analysis of prevalence (% of infected rodents), we applied maximum likelihood techniques based on log linear analysis. REGION of tick origin (three levels: eastern, western and gap regions; see Fig. 1), RODENT SPECIES (7 levels: *Apodemus agrarius, A. flavicollis, A. sylvaticus, Microtus arvalis, M. agrestis, A. oeconomus, Myodes glareolus*), SEX (males or females) and AGE (two levels: juvenile or adult, based on breeding status) were used as the factors in the models, with the presence or absence of ticks considered as a binary factor (0, 1) and referred to as presence/absence of TICK. For each level of analysis in turn, beginning with the most complex model, involving all possible main effects and interactions, those combinations which did not contribute significantly to explaining variation in the data were eliminated in a stepwise fashion beginning with the highest level interaction (backward selection procedure). A minimum sufficient model was then obtained, for which the likelihood ratio of chi-square was not significant, indicating that the model was sufficient to explain the data [19, 20].

General linear models (GLMs in SPSS v.21) were used for analysis of the mean tick abundance, using models with normal errors, incorporating the region of rodent origin, species, sex and age of rodents as fixed factors. Means are presented with the standard error of the mean (SE).

## Results

In total, 345 rodents were involved in the study: 132 *Apodemus* spp.: 77 striped field mice *A. agrarius*, 39 yellow-necked mice *A. flavicollis*, 16 wood mice *A. sylvaticus*; 158 root voles *A. oeconomus*; 47 *Microtus* spp.: 25 common voles *M. arvalis*, 22 field voles *M. agrestis* and 8 bank voles *M. glareolus*. Seven rodent species were trapped in the Eastern region, five in the Western region and four in the gap area (Additional file 1: Table S1).

Altogether, 5434 instars of three tick species were collected: 2866 *D. reticulatus* (2397 L and 469 N); 2141 *I. ricinus* (1957 L and 184 N) and 427 *H. concinna* (405 L and 22 N). In the region of the Eastern population, *D. reticulatus* was the dominant tick species collected from rodents (Additional file 1: Table S1, 2853 of juvenile ticks, 72.3%). In the region of the Western *D. reticulatus* population, *I. ricinus* was the most abundant tick species, followed by *H. concinna* and *D. reticulatus* (only 13 specimens collected). In the gap area, only *I. ricinus* instars were found on rodents (Additional file 1: Table S1).

Juvenile *D. reticulatus* were found in every site in the Eastern endemic region of *D. reticulatus* occurrence: in Białobrzegi (on 79.7% of 74 rodents), Stoski (76.2% of 21 rodents), Franciszków (71.4% of 14 rodents) and Urwitałt (60% of 50 rodents) and in one site in the Western region of *D. reticulatus* occurrence: Nowy Młyn (14% of 50 examined rodents). According to our expectations, in Niewiadów site (located in the gap area) no *D. reticulatus* instars were recorded during all trapping sessions. *Ixodes ricinus* was found in all trapping locations, and *H. concinna* was recorded in one site in the Western region of *D. reticulatus* occurrence (Nowy Młyn).

The overall prevalence of tick infestation was very high (88.4%). Among all examined hosts from all regions, the dominant species was *I. ricinus* (74.8% of infestation), followed by *D. reticulatus* (35.4%) and *H. concinna* (6.2%). Overall mean tick abundance differed among the three tick species (*D. reticulatus*: 8.32 ± 1.84 ticks/individual; *I. ricinus*: 6.21 ± 0.66 ticks/individual; *H. concinna*: 1.24 ± 0.51 ticks/individual); 60.3% of rodents harbored one tick species, and 28.1% were infested by two tick species.

### Prevalence of tick infestation by region

Total prevalence of ticks was slightly different among the three examined regions and was the highest in the Western region (three tick species present, 91.3%) and the lowest in the gap area (only *I. ricinus* noted, 80.7%); in the Eastern region it reached 90.6% (REGION × TICK presence/absence: *χ*^2^ = 5.74, *df* = 2, *P* = 0.06).

**Fig. 2:**
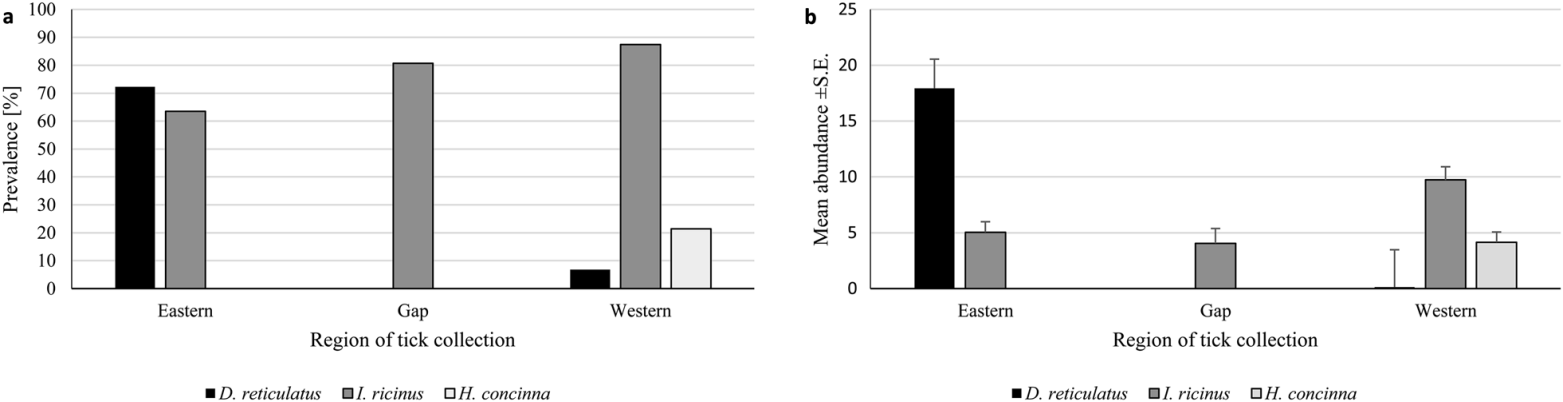
**a** Prevalence of tick species by region. **b** Mean abundance ± SE of tick species by region

Juvenile *I. ricinus* were collected in all sites in all examined regions; the highest infestation was recorded in the Western and the lowest in the Eastern region of the *D. reticulatus* populations (REGION × *I. ricinus* presence/absence: *χ*^2^ = 21.53, *df* = 2, *P* < 0.001; Fig. 2a).

Juvenile *D. reticulatus* were collected from rodents trapped in the areas of Eastern and Western *D. reticulatus* populations (infestation more than ten times higher in the Eastern region; Fig. 2a), but not from rodents trapped in the gap area (REGION × *D. reticulatus* presence/absence: *χ*^2^ = 209.54, *df* = 2, *P* < 0.001).

*Haemaphysalis concinna* (21.4%) were collected only from rodents trapped in the Western region of *D. reticulatus* occurrence (REGION × *H. concinna* presence/absence: *χ*^2^ = 56.83, *df* = 2, *P* < 0.001; Fig. 2a).

### Mean abundance of tick infestation by region

Overall mean abundance of ticks was high (15.75 ± 2.04 ticks/individual). The highest mean abundance was noted in rodents trapped in the Eastern region of *D. reticulatus* occurrence (> 20 ticks/individual; Additional file 1: Table S1) compared to rodents from the Western region (about 14 ticks/individual; Additional file 1: Table S1). Since only one tick species was present in the gap area (*I. ricinus*), the lowest mean abundance was recorded for rodents from that area, only 4.06 ± 1.32 (main effect of REGION on tick abundance: *F*_(2, 344)_ = 7.21, *P* < 0.001; Additional file 1: Table S1).

Mean abundance of juvenile *D. reticulatus* was > 100 × higher in the Eastern than in the Western region (main effect of REGION on *D. reticulatus* abundance: *F*_(2, 344)_ = 12.56, *P* < 0.001; Fig. 2b). In contrast, juvenile *I. ricinus* ticks were the most abundant in the Western region of *D. reticulatus* occurrence (Fig. 2b) with similar abundance in rodents from the Eastern region and the gap area (main effect of REGION on *I. ricinus* abundance: *F*_(2, 344)_ = 6.51, *P* = 0.01). Juvenile *H. concinna* was the second most abundant tick species in the Western region (main effect of REGION on *H. concinna*: *F*_(2, 344)_ = 7.20, *P* < 0.001; Fig. 2b).

### Co-infestation with different tick species by region

Since *I. ricinus* was the only tick species detected on rodents from the gap area, co-infestation could be observed only in the Eastern and Western regions. An identical percentage of examined rodents (45.3%) was infested with one (*I. ricinus* or *D. reticulatus*) or two tick species (*I. ricinus* + *D. reticulatus*) in the Eastern region. In the Western region, 67% of rodents were infested with one tick species, and 24.3% were infested with two tick species (*I. ricinus* + *D. reticulatus* or *I. ricinus* + *H. concinna*) (REGION × TICK CO-INFESTATION: *χ*^2^ = 78.71, *df* = 4, *P* < 0.001). Rodent species had no significant effect on occurrence of tick co-infestations (not significant, NS).

### Monthly activity of juvenile *Dermacentor reticulatus*

Instars of *D. reticulatus* were collected in each month of the study. The highest infestation with larvae was observed in June (56.4% of rodents), with a similar low prevalence in July and August (9.5% and 9.7%, respectively) (*D. reticulatus* LARVAE presence/absence × MONTH: *χ*^2^ = 47.16, *df* = 2, *P* < 0.001). The highest infestation with *D. reticulatus* nymphs was observed in July (38.1%), followed by August (29.0%) and June (25.6%) (*D. reticulatus* NYMPH presence/absence × MONTH: *χ*^2^ = 35.80, *df* = 2, *P* < 0.001).

### Effect of host species on tick infestation

All bank voles *M. glareolus* were infested with ticks (Additional file 1: Table S1). High prevalence of infestation (> 90%) was found on three species of *Apodemus* mice (Additional file 1: Table S1). Similarly, almost 90% of root voles *A. oeconomus* were tick-infested. Tick infestation of both *M. agrestis* and *M. arvalis* was slightly lower and similar (about 80%) (RODENT SPECIES × TICK presence/absence: *χ*^2^ = 14.90, *df* = 6, *P* = 0.02; Additional file 1: Table S1). Neither host age nor host sex had any significant effect on tick infestation (NS).

Juvenile *D. reticulatus* ticks were collected from all rodent species (7 species; Additional file 1: Table S1). Because of definitely higher prevalence in the endemic (Eastern) region of *D. reticulatus* compared to the Western region (only 13 specimens of *D. reticulatus* collected from rodents), we compared prevalence of *D. reticulatus* instars only for rodent species from the Eastern region. The highest *D. reticulatus* infestation (97.4%) was recorded in *A. oeconomus,* followed by *M. agrestis* (86.7%), *A. agrarius* (77.8%) and *M. glareolus* (71.4%). Lower prevalence of *D. reticulatus* infestation was detected in *M. arvalis* (68.4%), *A. sylvaticus* and *A. flavicollis* (66.7 and 65%, respectively) (RODENT SPECIES × *D. reticulatus* presence/absence: *χ*^2^ = 16.07, *df* = 6, *P* = 0.01; Additional file 1: Table S1).

Larvae and nymphs of *I. ricinus* were also collected from all seven host species, but the infestation was particularly high for *Apodemus* mice (Additional file 1: Table S1), the highest being for *A. flavicollis* (89.7%), followed by *A. sylvaticus* (87.5%) and *A. agrarius* (84.4%), and much lower for voles (*Microtus* + *Alexandromys*): *A. oeconomus* 72.8%, *M. arvalis* 56.0% and *M. agrestis* (45.5%), for *M. glareolus* 62.5% (RODENT SPECIES × *I. ricinus* presence/absence: *χ*^2^ = 24.98, *df* = 6, *P* < 0.001) in all regions (Additional file 1: Table S1).

Only three rodent species from the Western region of *D. reticulatus* occurrence harbored *H. concinna* ticks. The highest tick infestation was observed in *A. oeconomus*, followed by *M. agrestis* and *A. agrarius* (RODENT SPECIES × *H. concinna* presence/absence: *χ*^2^ = 23.50, *df* = 6, *P* = 0.01; Additional file 1: Table S1).

### Tick location on the host body

Some differences were observed in the location of ticks on the host body; however, this was based on general observations, not measured in exact numbers. About 75% of *I. ricinus* instars fed on the ear surface, between the toes and on the rodent’s head region. About 80% of *D. reticulatus* larvae and nymphs were found on the surface of the ears (larvae) and deep in the ear canals (nymphs), while about 90% of juvenile *H. concinna* were found on the ventral site of the rodent body.

## Discussion

In the current study we supported the results of our previous study on questing ticks: both larvae and nymphs of *D. reticulatus* were found on rodents from the regions of the Western and Eastern tick populations, and no juvenile ticks were recorded at the site located in the gap area. This finding confirms the existence of two stable populations of ornate dog ticks, including the areas recently invaded by this tick species (expansion zones; [5, 6]).

Furthermore, very high mean infestation of rodents trapped in the Eastern region of *D. reticulatus* occurrence by juvenile stages of *D. reticulatus* was in agreement with extremely high mean abundance of adult ticks recorded previously in Białobrzegi (91 ticks/100 m^2^), Urwitałt (44 ticks/100 m^2^) and Stoski (50.5 ticks/100 m^2^ [6]).

Interestingly, the share of *D. reticulatus* in the tick community of rodents was reversed in two regions, as *D. reticulatus* instars constituted the majority of ticks collected from rodents in the region of the Eastern population and were the least common/abundant among the tick community in Western Poland, where *H. concinna* was more common/abundant than *D. reticulatus* [7]. In one of the sites in the Western region (Słonin) we did not detect larvae and nymphs of *D. reticulatus*, although it was located about 40 km from the outer border of the *D. reticulatus* Western population and only 8–10 km from the nearest site positive for adult *D. reticulatus* [5, 6]. In Nowy Młyn site adult *D. reticulatus* were collected in spring 2018 with a density of 9.50 ticks/100 m^2^ [6], but then we collected only 10 *D. reticulatus* nymphs from 39 rodents in the summer months of instar activity [7]. To the best of our knowledge, this present study is also the first to report the occurrence of juvenile *D. reticulatus* ticks in Western Poland; to date only adult questing *D. reticulatus* ticks have been sampled in that region [21, 22].

Again, this region-dependent structure of the rodent tick community reflects ideally the dominance of adult *D. reticulatus* among ticks collected from hosts in the region of the Eastern tick metapopulation (in both Poland [23] and Ukraine [24]). The low share of this tick species in the tick community of different rodent species was also observed in the Western metapopulation: in Germany [25–28] and The Netherlands [9], which is an interesting repeatable observation of unknown reason. In the current study, the lack of *D. reticulatus* in the gap area and high prevalence/abundance in the region of the Eastern population resulted in 100 × higher total abundance of ticks on rodents from this region. Thus, the gap area, historically free of *D. reticulatus*, was also characterized by the very low total tick abundance on rodents. However, in view of dynamic expansion of *D. reticulatus* populations in the area of Poland [6], we may expect that the gap area will be colonized by these ticks within a 10-year period.

It is worth underlining that our trapping sites were located in open habitats to focus trapping efforts on *Microtus*/*Alexandromys* voles, predicted as the main hosts for juvenile *D. reticulatus* [7, 8, 12, 13]. However, the selected sites were also rich in vegetation (plant cover up to 1.5 m) and humid even in the hot summer period (submerged meadows, surface water borders). As a result, we sampled a rich rodent community comprising both species typical for open habitats (*Microtus*/*Alexandromys* voles, *A. agrarius*) and ecotone/woodland species (*A. sylvaticus*, *M. glareolus*, *A. flavicollis*). We think that this species-rich rodent community together with suitable humidity conditions of the habitats resulted in the occurrence of three tick species, including species with high humidity requirements (*I. ricinus*, *H. concinna*) [2, 29, 30]. In our previous study in forest sites in NE Poland, *I. ricinus* instars were clearly dominant in the tick community of woodland rodents and *D. reticulatus* was dominant only among ticks collected from the common vole, *M. arvalis*, which prefers less humid habitats than the root vole [13, 31]. Our study revealed also that a wide range of open habitats or woodland rodents may constitute suitable hosts for juvenile *D. reticulatus* (*n* = 7, present study). As all these rodent species are common and widespread in the whole area of Poland/Europe, they can easily support the settlement of new *D. reticulatus* populations following the transportation/introduction of engorged females by wildlife (i.e. cervids, wild boar [32] or dogs traveling with their owners [33] and contribute to fast expansion of this tick species.

As both *D. reticulatus* and *I. ricinus* instars were observed on rodent ears (current study), this co-occurrence may have resulted in co-feeding [34] and predisposed to the exchange of pathogens between these two species. This phenomenon may have important consequences as both tick species are competent vectors of tick-borne encephalitis virus (TBEV) [4, 35, 36]. The majority of these rodent species are known as a TBEV reservoir hosts [37], and co-feeding has been found to enable virus transmission in an experimental study [38]. For these reasons, co-feeding may increase the density of TBEV-infected *I. ricinus* as well as *D. reticulatus* in such habitats, bringing enhanced health risks.

Influence of co-feeding of *D. reticulatus* and *I. ricinus* on *Borrelia burgdorferi*-infected rodents cannot be simply predicted, as ticks from genus *Dermacentor* were not experimentally confirmed as vectors of any *B. burgdorferi* (s.l.) spirochaetes [39, 40]. It would be interesting to investigate whether the prevalence of *Borrelia burgdorferi* (s.l.) is lower in adult *I. ricinus* ticks in such habitats, where *D. reticulatus*' contribution to the tick community in rodents is very high and may have resulted in a type of ‘dilution effect’ [41].

Our results about seasonality of larvae and nymphs of *D. reticulatus* are similar to other studies regarding the activity of *D. reticulatus* instars [14, 15]. Most of the larvae were collected in June, and the peak of activity of nymphs was noted in July. However, due to the emerging climate changes we can expect changes in seasonal tick activity [42].

During current and previous studies on ticks collected from the environment we did not collect juvenile *D. reticulatus* from vegetation on any occasion. Additionally, juvenile ticks of this species were not collected from medium-sized mammals, i.e. domestic dogs and red foxes, hosts of adult ticks [20, 23, 43]. Interestingly, recently few *D. reticulatus* nymphs were collected by the flagging method from vegetation during the highest peak activity of instars in Germany [44]. However, this may be more of an exceptional situation than a common phenomenon and needs to be verified.

## Conclusions

We demonstrated that the presence of juvenile *D. reticulatus* on rodents from open habitats reflects the distribution of adult ticks and can be used as an indicator of the existence of a stable tick population. We have also demonstrated the wide range of rodents as important hosts for juvenile ticks, including *D. reticulatus*, and the great contribution of this tick species to the tick community in endemic regions, especially in the area of the Eastern tick population/metapopulation. Finally, co-infestation of *I. ricinus* and *D. reticulatus* can result in co-feeding and may affect the circulation of pathogens of medical significance in such regions.

## Supplementary Information

**Additional file 1: Table S1**. Tick prevalence and mean abundance by host species by region; nt: number of ticks collected.

## Data Availability

All data generated or analysed during this study are included in this published article.

## Abbreviations

L: Larvae
N: Nymphs
NS: Not significant
SE: Standard error
TBEV: Tick-borne encephalitis virus
