# Supplementary material for: Occurrence of juvenile Dermacentor reticulatus ticks in three regions in Poland: the final evidence of the conquest
Source: Parasit Vectors. 2021 Oct 14;14:536. doi: 10.1186/s13071-021-05039-z (PMC8518239; doi:10.1186/s13071-021-05039-z)
Supplement: Supplementary file 1 — Additional file 1: Table S1. Tick prevalence and mean abundance by host species by region; nt: number of ticks collected. [file 13071_2021_5039_MOESM1_ESM.docx]

|  | Eastern region | | | | | | | | Gap region | | | | Western region | | | | | | | | | | |
| --- | --- | --- | --- | --- | --- | --- | --- | --- | --- | --- | --- | --- | --- | --- | --- | --- | --- | --- | --- | --- | --- | --- | --- |
|  |  | *D. reticulatus* | | | *I. ricinus* | | |  | *Ixodes ricinus* | | | |  | *D. reticulatus* | | | *I. ricinus* | | | *H. concinna* | | |  |
| Rodents species | No of rodents | Larvae (nt)  % infested  Mean +/- SE | Nymphs (nt) % infested  Mean +/- SE | Total  (nt) % infested  Mean +/- SE | Larvae (nt) % infested  Mean +/- SE | Nymphs  (nt) % infested  Mean +/- SE | Total  (nt) % infested  Mean +/- SE | Total ticks (nt) % infested  Mean +/- SE | No of rodents | Larvae (nt) % infested  Mean +/- SE | Nymphs (nt) % infested  Mean +/- SE | Total ticks (nt) % infested  Mean +/- SE | No of rodents | Larvae (nt)  % infested  Mean +/- SE | Nymphs  (nt) % infested  Mean +/- SE | Total  (nt)  % infested  Mean +/- SE | Larvae  (nt) % infested  Mean +/- SE | Nymphs  (nt) % infested  Mean +/- SE | Total  (nt) % infested  Mean +/- SE | Larvae  (nt) % infested  Mean +/- SE | Nymphs  (nt) % infested  Mean +/- SE | Total  (nt) % infested  Mean +/- SE | Total ticks (nt) % infested  Mean +/- SE |
| *Apodemus agrarius* | 38 | 52.6% 12.13± 5.08  nt=461 | 39.5% 0.97±0.73  nt=37 | 73.7% 13.11±5.32 nt=  498 | 63.2% 4.91±1.79 nt=187 | 23.7% 0.47±0.32 nt=18 | 71.1% 5.40±1.92 nt=205 | 94.7% 18.50±6.06 nt=703 | 17 | 100% 7.47±2.68 nt=127 | 17.6% 0.24±0.47 nt= 4 | 100% 7.71±2.87  nt=131 | 22 | 0 | 4.5% 0.05±0.96  nt=1 | 4.5%. 0.05±0.99 nt=1 | 95.5% 9.09±2.36  nt=200 | 36.4% 0.50±0.42 nt= 11 | 95.5%. 9.59±2.52 nt=211 | 4.5% 0.05±1.78  nt=1 | 0 | 4.5% 0.05±1.97  nt=1 | 95.5% 9.68±7.96 nt=212 |
| *A. flavicollis* | 21 | 61.9% 8.76±6.83  nt=184 | 4.8% 0.10±0.98 nt=  2 | 61.9% 8.86±7.16 nt=  186 | 90.5%  10.67±2.41  nt=  224 | 19%  0.27±0.43 nt=  6 | 90.5% 10.95±2.60  nt= 230 | 95.2% 19.81±8.15 nt=416 | 4 | 100% 5.50±5.52 nt=22 | 0 | 100% 5.50±5.91  nt=22 | 14 | 0 | 0 | 0 | 85.7% 13.36±2.95 nt=  187 | 42.9% 1.14±0.53 nt=16 | 85.7% 14.50±3.16  nt= 203 | 0 | 0 | 0 | 85.7% 14.50±9.98 nt=  203 |
| *A. sylvaticus* | 10 | 50%.  7.30±9.90 nt=73 | 10% 0.1±0.42 nt=1 | 60% 7.40±10.40  nt=74 | 80% 14.30±3.49  nt=143 | 0 | 80% 14.30±3.74 nt=143 | 90% 21.70±11.80  nt=217 | 0 | 0 | 0 | 0 | 6 | 0 | 0 | 0 | 100% 15.50±4.51 nt=  93 | 0 | 100% 15.50±4.83 nt=93 | 0 | 0 | 0 | 100% 15.50±14.24  93 |
| Total *Apodemus* | 69 | 55.1% 10.41±3.77  nt=718 | 24.6%  0.58. ±0.57 nt=40 | 68.1% 10.99±3.97 nt=  758 | 73.9% 8.03± 1.33 nt=554 | 18.8% 0.35±0.23 nt=24 | 78.3% 8.38±1.42 nt=578 | 94.2% 19.36±4.50 nt=1336 | 21 | 100% 7.10±2.42 nt=  149 | 14.3% 0.19±0.42 nt=4 | 100% 7.29±2.58 nt=  153 | 42 | 0 | 2.4% 0.02±0.72 nt=1 | 2.4% 0.02±0.72  nt=1 | 92.9% 11.43±1.71 nt=  480 | 33.3%  0.64±0.3 0 nt=27 | 92.9% 12.07±1.83 nt=  507 | 2.4% 0.02±1.28 nt=1 | 0 | 2.4% 0.02±1.41 nt=1 | 92.9% 12.12±5.77 nt=508 |
| *Alexandromys oeconomus* | 40 | 62.5% 28.08±4.95  nt=1123 | 75% 8.3±0.71 nt=  321 | 92.5% 36.10±5.18 nt=  1444 | 42.5% 2.08±1.75 nt=  83 | 12.5% 0.25±0.31 nt=10 | 50% 2.33±1.87 nt=93 | 95% 38.43±5.90  nt=1537 | 61 | 75% 2.25±1.41 nt=137 | 24.6% 0.72±0.23 nt=44 | 73.8% 2.98±1.51  nt=181 | 57 | 1.8% 0.02±4.15  nt=1 | 10.5% 0.019±0.60 nt=11 | 10.5% 0.21±4.34  nt=12 | 86% 7.63±1.46 nt=435 | 26.3% 074±0.29 nt=42 | 87.7% 8.37± 1.57  nt=477 | 33.3% 6.90±1.11 nt=393 | 5.3% 0.39±0.31  nt=22 | 33.3% 7.28±1.23 nt=415 | 93% 15.86±4.94 nt=905 |
| *Microtus arvalis* | 25 | 32% 15.08±6.23 nt=377 | 28% 1.54±0.90  nt=41 | 52% 16.72±6.56 nt=  418 | 48% 1.28±2.21 nt=  32 | 16% 0.88±0.39 nt=  22 | 56% 2.16±2.36 nt=54 | 76% 18.88±7.47 nt=472 | 0 | 0 | 0 | 0 | 0 | 0 | 0 | 0 | 0 | 0 | 0 | 0 | 0 | 0 | 0 |
| *M. agrestis* | 18 | 27.8% 4.06±7.38 nt=73 | 61.1% 2.94±1.96 nt=53 | 72.2% 7.00±7.23  nt=126 | 50% 1.83±2.60  nt=33 | 11.1% 0.39±0.46 nt=7 | 50% 2.22±2.77 nt=40 | 83.3% 9.22±8.80 nt=166 | 0 | 0 | 0 | 0 | 4 | 0 | 0 | 0 | 25% 4.50±5.52 nt=18 | 0 | 25% 4.50±5.90  nt= 18 | 50% 2.75±4.18 nt=11 | 0 | 50% 2.75±4.63 nt=11 | 50% 7.25±18.66 nt=29 |
| Total *Microtus*+  *Alexandromys* | 87 | 45.8% 18.92±3.44 n=1573 | 57.8% 5.00±0.52 nt=415 | 75.9% 23.95±3.62 nt=  1988 | 45.8% 1.78±1.22 nt=148 | 13.3% 0.47±0.21 nt=39 | 51.8% 2.53±1.30 nt=187 | 86.7% 26.21±4.11 nt=2175 | 61 | 70.5% 2.25±1.42 nt=137 | 24.6% 0.72±0.25 nt=44 | 73.8% 2.58±1.51 nt=181 | 61 | 1.6% 0.02±4.01  nt=1 | 9.8% 0.18±0.80 nt=12 | 9.8% 0.19±4.22  nt=13 | 82% 7.43±1.42 nt=453 | 24.6% 0.69±0.25 nt=42 | 24.6% 5.29±4.47  nt=494 | 34.4% 6.23±1.06 nt=404 | 4.9% 0.36±0.13 nt=22 | 34.4% 6.98±1.19 nt=426 | 90.2% 15.30±4.79 nt=934 |
| *Myodes glareolus* | 7 | 42.9% 15.00±11.83 nt=105 | 28.6% 0.29±1.70  nt=2 | 71.4% 15.23±12.39  nt=107 | 57.1% 4.86±4.18  nt=34 | 14.3% 0.43±0.76 nt=3 | 57.1% 5.29±4.47 nt=37 | 100% 20.57±14.11  nt=144 | 1 | 100% 2.00±11.05 nt=1 | 100% 2.00±11.05  nt=1 | 100% 3.00±11.82 nt=2 | 0 | 0 | 0 | 0 | 0 | 0 | 0 | 0 | 0 | 0 | 0 |
| Total rodents | 159 | 49.7% 15.07±2.47  nt=2396 | 42.1% 2.87± 0.39 nt=457 | 72.3% 17.93±2.62 nt=2853 | 58.5% 4.63±0.90 nt=736 | 15.7% 0.42±0.15 nt=66 | 63.5% 5.04±0.95 nt=802 | 90.6% 22.99±2.95 nt=3655 | 83 | 78.3% 3.47±1.24 nt=288 | 22.9% 0.59±0.21 nt=49 | 80.7% 4.06±1.32 nt=337 | 103 | 1% 0.01. ±3.47 nt=1 | 6.8% 0.12±0.48 nt=12 | 6.8% 0.13±3.25 nt=13 | 86.4% 9.06±1.11 nt=933 | 28.2% 0.61±0.19 nt=69 | 87.4% 9.73±1.18 nt=1002 | 21.4% 3.93±0.83 nt=405 | 2.9% 0.21±0.10 nt=22 | 21.4% 4.15±0.92 nt=427 | 91.3% 14.00±3.67 nt=1442 |

Additional file 1: Table S1. Tick prevalence and mean abundance by host species by region; nt- number of tick collected
